# Influence of *Trichosanthes pericarpium* extract on improving microcirculation and outcomes of patients with acute myocardial infarction after percutaneous coronary intervention

**DOI:** 10.3389/fcvm.2023.1126573

**Published:** 2024-01-04

**Authors:** Peng Xi, Yuan Xie, Feifei Huang, Yang Liu, Jiahong Xu

**Affiliations:** Department of Cardiology, Shanghai Tongji Hospital, Tongji University School of Medicine, Shanghai, China

**Keywords:** acute myocardial infarction, microcirculation, percutaneous coronary intervention, Chinese traditional herb, outcomes

## Abstract

**Background:**

Microcirculatory dysfunction is an independent risk factor for a poor prognosis after percutaneous coronary intervention (PCI) in patients with acute myocardial infarction (AMI). *Trichosanthes pericarpium* is a well-known Chinese traditional herb described with the effect of activating blood circulation to dissipate blood stasis and improve blood circulation. However, its effects on microcirculation in patients with AMI after primary PCI remain unknown. This study aimed to investigate the effect of *Trichosanthes pericarpium* extract (TPE) on improving microcirculation and outcomes in patients with AMI after PCI.

**Methods:**

This study included 155 patients with a history of emergency PCI treatment. In this cohort, 31 patients received a course of TPE, defined as intravenous TPE infusion (12 ml TPE dissolved in 250 ml 5% Glucose Injection) once daily for 7 days on the background of standard treatment after PCI; 124 who did not receive TPE were regarded as the control group and received standard treatment after PCI. The corrected thrombolysis in myocardial infarction frame count (CTFC) and index of microcirculatory resistance (IMR) were used to evaluate myocardial microcirculation. Cardiac function was measured by echocardiography during hospitalization and follow-up. Major adverse cardiac events (MACEs) were recorded for prognostic analysis.

**Results:**

At the 6-month follow-up, AMI patients who received TPE after primary PCI had significantly lower levels of CTCF (24.27 ± 2.40 vs. 21.88 ± 1.92, *P* < 0.001) and IMR (20.02 ± 2.20 vs. 17.80 ± 2.11, *P* < 0.001) than patients in the control group. Left ventricular ejection fraction and left ventricular internal dimension at systolic at 6-month follow-up in the TPE group significantly improved than in the control group (56.6 ± 4.5 vs. 62.1 ± 3.5, *P* < 0.001; 32.5 ± 1.5 vs. 30.2 ± 1.8, *P* < 0.001). Kaplan-Meier curve analysis indicated that patients with AMI who received TPE had significantly lower rates of MACEs than the control group at 6-month follow-up (*P* = 0.042).

**Conclusion:**

In the context of standard treatment, *Trichosanthes pericarpium* further improved coronary microcirculation, increased cardiac function, and reduced short-term MACEs rate. Our data suggest that TPE could be used in combination therapy for patients with AMI after primary PCI.

## 1 Introduction

Cardiovascular diseases, particularly acute myocardial infarction (AMI), remain a leading cause of adult morbidity and mortality worldwide (1, 2). Although AMI is caused by coronary artery obstruction, the coronary microcirculation is crucial for survival and recovery of local myocardium after AMI and also plays an important role in the development, course and prognosis of the disease (3). Over the past few decades, advances in catheter-based reperfusion have improved outcomes in patients with AMI. However, it has been reported that 20%–60% of patients still experience recurrent angina after percutaneous coronary intervention (PCI) due to coronary microcirculatory dysfunction (4). Meanwhile, restoration of coronary blood flow does not represent effective perfusion of myocardial microcirculation, and coronary microcirculatory dysfunction has been shown to increase the risk of cardiovascular events and worsen the prognosis of patients (5–7). Therefore, it is an urgent need to explore effective therapeutic means to improve myocardial microcirculation perfusion in AMI patients after PCI.

*Trichosanthes pericarpium* extract (TPE) is derived from the dried mature pericarp of *Trichosanthes kirilowii* Maxim and *Trichosanthes rosthornii* Harms. It is a well-known Chinese traditional herb, rich in polysaccharides, flavonoids and amino acids and other chemical components, described with the effect of anti-inflammatory, anti-oxidation, regulating lipid metabolism, activating blood circulation to dissipate blood stasis and improve blood circulation. TPE has protective effects against inflammation, ischemia-reperfusion injury, angina, and various cardiovascular diseases (8–10). It has been reported that TPE promotes the mobilization of endothelial progenitor cells by up-regulating the expression levels of vascular endothelial growth factor, endothelial nitric oxide synthesis, nitric oxide and matrix metalloproteinase 9, resisting myocardial injury induced by AMI (11). In addition, TPE can also inhibit myocardial apoptosis induced by hypoxia/reoxidation injury by activating the phosphatidylinositol 3-kinase/protein kinase B signaling pathway (12). However, its influence on microcirculation after direct PCI and the outcomes in patients with AMI remain unknown.

In the present study, we enrolled 31 patients with AMI who received TPE before and after PCI and 124 who did not receive TPE as matched controls. This study aimed to provide more evidence for applying traditional Chinese herbal extracts in therapeutic strategies for patients with AMI.

## 2 Materials and methods

### 2.1 Study population

**Figure 1:**
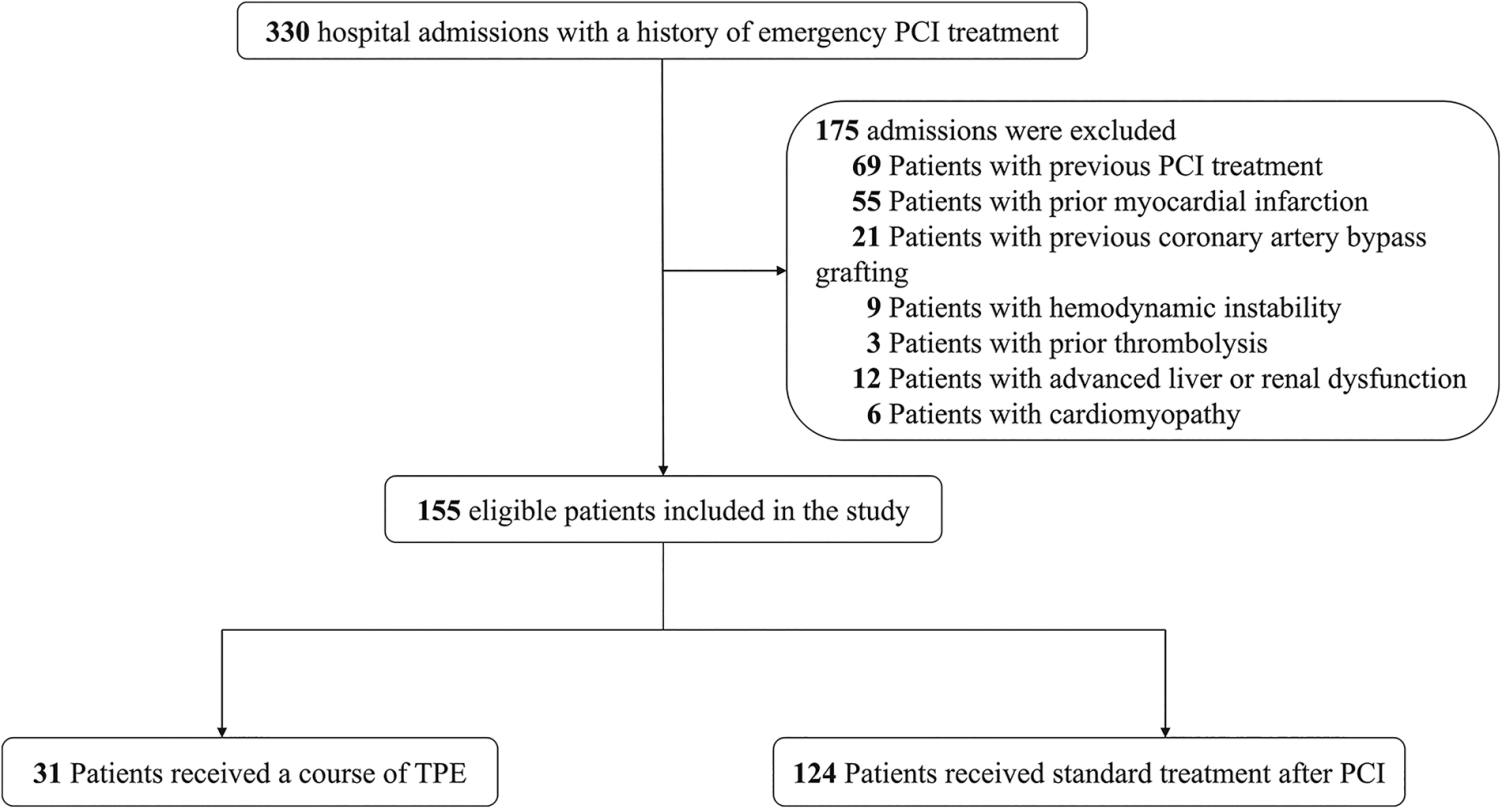
Study design.

From March 2019 to September 2021, 155 patients with AMI admitted to the Department of Cardiology of Tongji University Affiliated Tongji Hospital with a history of emergency PCI treatment were retrospectively included in this study. All patients enrolled in the study underwent angiography for primary PCI and follow-up angiography 6 months after the PCI. The exclusion criteria were as follows: (1) previous PCI, (2) prior myocardial infarction, (3) previous coronary artery bypass grafting, (4) hemodynamic instability, (5) prior thrombolysis, (5) advanced liver or renal dysfunction, and (6) cardiomyopathy (Figure 1).

### 2.2 Study design

In this Study, 31 patients were administered TPE, defined as intravenous TPE infusion (12 ml TPE dissolved in 250 ml 5% Glucose Injection) once daily for 7 days after PCI on the background of standard treatment; 124 patients with AMI who did not receive TPE were regarded as the control group and received standard treatment after PCI. TPE used in this study was manufactured by Shanghai pharma No.1 Biochemical & Pharmaceutical Co., Ltd. Major adverse cardiac events (MACEs) were defined as recurrent MI, new heart failure, intractable angina, and cardiac death. Clinical data were retrospectively reviewed according to the records, including age, female sex, body mass index (BMI), blood pressure, heart rate, medical history, and drug intervention. The high sensitivity C-reactive protein (hs-CRP) level, neutrophil to lymphocyte ratio (NLR) and endothelin-1 level were also recorded. This study was approved by the Ethics Committee of Tongji Hospital, Tongji University. Written informed consent was obtained from all patients to allow for the analysis of clinical information for scientific research purposes.

### 2.3 Coronary angiography parameters

Coronary angiography was performed using standard techniques. All angiograms from the internal and external diagnostic cohorts were recorded at 30 FPS and analyzed by two independent cardiologists blinded to the patient clinical data. The corrected thrombolysis in myocardial infarction (TIMI) frame count (CTFC) and index of microcirculatory resistance (IMR) were obtained from the coronary angiography of the primary PCI and at a 6-month follow-up after primary PCI. The CTFC was calculated as the number of frames required for the dye to reach standardized distal landmarks at 30 FPS. The first frame was defined as the frame when the dye fully entered the artery, whereas the last frame was defined as the frame at which the dye entered the branch of the distal landmark. Since the TIMI frame count of the left anterior descending artery (LAD) is approximately 1.70 times that of the left circumflex artery (LCX) and the right coronary artery (RCA), the longer LAD frame count was corrected by dividing it by 1.70 to obtain the CTFC. The IMR measurement was conducted as previously described using a commercial software (FlashAngio, Rainmed Ltd, Suzhou, China).

### 2.4 Echocardiography

Echocardiography was performed at 7 days and 6 months after PCI by two independent sonographers blinded to the patient clinical data. The left ventricular ejection fraction (LVEF), left ventricular internal dimension at systolic (LVIDs), left ventricular internal dimension at diastole (LVIDd), interventricular septal thickness (IVST), and left ventricular posterior wall dimension (LVPWd) were obtained using anatomical M-mode echocardiography.

### 2.5 Statistics analysis

All statistical analyses were performed using IBM SPSS 22.0 (IBM Corp, Armonk, NY, USA). Continuous variables were presented as the mean difference ± standard deviation. The comparability of the characteristics between the two groups was assessed using the Student's *t*-test for continuous quantitative variables and the chi-square test for qualitative variables. Statistical significance was set at *P* < 0.05.

## 3 Results

### 3.1 Baseline characteristics and coronary lesion characteristics

This study enrolled 155 patients with AMI, of which 31 received TPE after primary PCI and 124 did not. The baseline characteristics of the patients are shown in Table 1, and the detailed coronary lesion characteristics obtained by cardio angiography are shown in Table 2. As shown in Table 1, there were no significant differences in sex, age, heart rate, blood pressure, BMI, medical history, or drug intervention between the two groups (all *P* > 0.05). The door-to-balloon time, the number of lesion arteries, main lesion artery, TIMI flow grade 3, and size and number of implanted stents between the two groups also showed no significant difference, as shown in Table 2 (all *P* > 0.05).

**Table 1:** Baseline characteristics.

	Total	Control group	TPE group	
*N*	155	124 (1.67%)	31 (48.33%)	
Female	73 (47.10%)	59 (47.58%)	14 (45.16%)	*χ*^2^ = 0.058 *P* = 0.809
Age	55.43 ± 10.10	55.56 ± 11.10	54.93 ± 10.08	*P* = 0.779
Heart rate	88.33 ± 16.76	88.11 ± 16.93	89.19 ± 16.02	*P* = 0.750
Systolic pressure	123.19 ± 15.70	123.15 ± 15.55	123.35 ± 16.25	*P* = 0.949
Diastolic pressure	63.83 ± 8.78	63.81 ± 9.54	63.90 ± 4.65	*P* = 0.957
BMI	21.12 ± 1.42	21.08 ± 1.04	21.29 ± 1.02	*P* = 0.312
Hypertension	89 (57.42%)	73 (58.87%)	16 (51.61%)	*χ*^2^ = 0.534 *P* = 0.465
Diabetes	79 (50.97%)	64 (51.61%)	15 (48.39%)	*χ*^2^ = 0.004 *P* = 0.950
Smoke	54 (34.84%)	44 (35.48%)	10 (32.26%)	*χ*^2^ = 0114 *P* = 0.736
cTnI max	21.22 ± 12.62	20.61 ± 11.12	23.65 ± 17.18	*P* = 0.234

BMI, body mass index; cTnI max, max cardiac troponin I; TPE, *Trichosanthes pericarpium* extract. Data are presented as mean ± SD.

**Table 2:** Coronary angiography parameters.

	Control group	TPE group	
Artery lesion	1.76 ± 0.77	1.74 ± 0.76	*P* = 0.917
LAD	79 (63.71%)	19 (61.29%)	*χ*^2^ = 0.062 *P* = 0.803
LCX	75 (60.48%)	19 (61.29%)	*χ*^2^ = 0.007 *P* = 0.934
RCA	62 (50.00%)	16 (51.61%)	*χ*^2^ = 0.026 *P* = 0.872
Stent number	1.96 ± 0.79	1.94 ± 0.80	*P* = 0.880
Stent diameter	2.87 ± 0.30	2.98 ± 0.35	*P* = 0.107
Stent length	26.95 ± 6.84	26.81 ± 6.11	*P* = 0.915
TIMI grade 3	105 (84.68%)	26 (83.87%)	*χ*^2^ = 0.012 *P* = 0.912

LAD, left anterior descending; LCX, left circumflex; RCA, right coronary artery; TPE, *Trichosanthes pericarpium* extract.

Data are presented as mean ± SD.

### 3.2 TPE improves cardiac microcirculation of AMI patients 6-months after primary PCI

To explore the potential effects of TPE on cardiac microcirculation in patients with AMI after primary PCI, we analyzed CTFC and IMR in both groups immediately after the procedure and at the 6-month follow-up. As shown in Table 3, there was no significant difference in CTFC and IMR immediately after the primary PCI between the two groups (all *P* > 0.05). The 6-month follow-up showed that CTFC (26.40 ± 2.98 vs. 24.27 ± 2.40, *P* < 0.001; 26.60 ± 4.47 vs. 21.88 ± 1.92, *P* < 0.001) and IMR (23.41 ± 1.38 vs. 20.02 ± 2.20, *P* < 0.001; 23.47 ± 1.55 vs. 17.80 ± 2.11, *P* < 0.001) were significantly decreased in both groups while patients with AMI who received TPE after primary PCI had significantly lower levels of CTCF (24.27 ± 2.40 vs. 21.88 ± 1.92, *P* < 0.001) and IMR (20.02 ± 2.20 vs. 17.80 ± 2.11, *P* < 0.001) than the control group.

**Table 3:** Comparison of microcirculatory assessment between the two groups after treatment.

	CTFC		IMR	
	Immediate postoperative	Follow up at 6-month	Immediate postoperative	Follow up at 6-month
Control group	26.39 ± 2.98	24.27 ± 2.40*	23.41 ± 1.38	20.02 ± 2.20*
TPE group	26.60 ± 4.47	21.88 ± 1.92*^,^^#^	23.47 ± 1.55	17.80 ± 2.11*^,^^#^

CTFC, corrected TIMI frame count; IMR, index of microcirculatory resistance; TPE, *Trichosanthes pericarpium* extract.

Data are presented as mean ± SD.

* *P* < 0.05 compared to immediate postoperative.

^#^ *P* < 0.05 compared to Control group.

### 3.3 TPE preserves the cardiac function of patients with AMI 6-months after primary PCI

To further examine the effects of TPE on cardiac function in patients with AMI after primary PCI, we analyzed echocardiographic parameters at 7 days and 6 months after primary PCI. As shown in Table 4, the LVIDs, LVIDd, IVST, LVPWd, and LVEF were comparable between the two groups 7 days after treatment. LVIDs were found to decrease significantly (35.6 ± 1.2 vs. 32.5 ± 1.5, *P* < 0.001; 35.4 ± 1.9 vs. 30.2 ± 1.8, *P* < 0.001) while LVEF was found to increase significantly in both groups at 6 months after treatment (53.7 ± 4.7 vs. 56.6 ± 4.5, *P* < 0.001; 53.3 ± 4.0 vs. 62.1 ± 3.5, *P* < 0.001). Compared to the echocardiography at 7 days, IVST and LVPWd at 6 months after treatment showed a trend to incrassation in the control group (9.6 ± 0.3 vs. 9.9 ± 0.4, *P* < 0.001; 9.6 ± 0.3 vs. 9.9 ± 0.5, *P* < 0.001) and had no significant change in the TPE group (9.6 ± 0.4 vs. 9.7 ± 0.4, *P* = 0.251; 9.5 ± 0.3 vs. 9.7 ± 0.2, *P* = 0.056), while LVIDd were found to be significantly decreased in the TPE group (45.4 ± 2.8 vs. 44.0 ± 1.5, *P* = 0.019) and had no significant change in the control group (45.0 ± 2.9 vs. 44.6 ± 3.0, *P* = 0.244). LVEF and LVIDs at 6 months after treatment in the TPE group significantly improved compared to those in the control group (56.6 ± 4.5 vs. 62.1 ± 3.5, *P* < 0.001; 32.5 ± 1.5 vs. 30.2 ± 1.8, *P* < 0.001). Moreover, LVPWd in the TPE group was thinner than in the control group (9.9 ± 0.5 vs. 9.7 ± 0.2, *P* = 0.008).

**Table 4:** Comparison of echocardiography between the two groups after treatment.

	LVIDs/mm		LVIDd/mm		IVST/mm		LVPWd/mm		LVEF/%	
	7 days	6 months	7 days	6 months	7 days	6 months	7 days	6 months	7 days	6 months
Control group	35.6 ± 1.2	32.5 ± 1.5*	45.0 ± 2.9	44.6 ± 3.0	9.6 ± 0.3	9.9 ± 0.4*	9.6 ± 0.3	9.9 ± 0.5*	53.7 ± 4.7	56.6 ± 4.5*
TPE group	35.4 ± 1.9	30.2 ± 1.8*^,^^#^	45.4 ± 2.8	44.0 ± 1.5*	9.6 ± 0.4	9.7 ± 0.4	9.5 ± 0.3	9.7 ± 0.2^#^	53.3 ± 4.0	62.1 ± 3.5*^,^^#^

LVIDs, left ventricular internal dimension at systolic; LVIDd, left ventricular internal dimension at diastole; IVST, interventricular septal thickness; LVPWd, left ventricular posterior wall dimension; LVEF, left ventricular ejection fraction; TPE, *Trichosanthes pericarpium* extract.

Data are presented as mean ± SD.

* *P* < 0.05 compared to 7 days.

^#^ *P* < 0.05 compared to Control group.

### 3.4 TPE inhibits inflammation and improves endothelial function of AMI patients 7-days after primary PCI

To explore the effects of TPE on microcirculatory inflammation and endothelial function, we analyzed hs-CRP, NLR and ET-1 in both groups immediately after the procedure and at 7 days after primary PCI. As shown in Table 5, there were no significant differences in hs-CRP, NLR and ET-1 between the two groups immediately after the procedure (all *P* > 0.05). AMI patients who received TPE for 7-days after PCI showed significantly lower level of hs-CRP, NLR and ET-1 than the control group (62.5 ± 24.1 vs. 21.3 ± 10.0, *P* < 0.001; 71.7 ± 7.4 vs. 60.3 ± 6.3, *P* < 0.001; 67.1 ± 11.5 vs. 50.1 ± 8.4, *P* < 0.001). Furthermore, the level of hs-CRP, NLR and ET-1 at 7-days in the TPE were found to be significantly decreased compared to those in the control group (21.3 ± 10.0 vs. 13.8 ± 9.1, *P* < 0.001; 60.3 ± 6.3 vs. 55.9 ± 5.5, *P* = 0.001; 50.1 ± 8.4 vs. 44.0 ± 9.1, *P* < 0.001).

**Table 5:** Comparison of inflammation assessment between the two groups after treatment.

	Hs-CRP		NLR		ET-1	
	Immediate postoperative	7 days	Immediate postoperative	7 days	Immediate postoperative	7 days
Control group	62.5 ± 24.1	21.3 ± 10.0*	71.7 ± 7.4	60.3 ± 6.3*	67.1 ± 11.5	50.1 ± 8.4*
TPE group	61.9 ± 29.2	13.8 ± 9.1*^,^^#^	71.6 ± 7.4	55.9 ± 5.5*^,^^#^	67.3 ± 12.1	44.0 ± 9.1*^,^^#^

Hs-CRP, hypersensitive C-reactive protein; NLR, neutrophil to lymphocyte ratio; ET-1, endothelin-1; TPE, *Trichosanthes pericarpium* extract.

Data are presented as mean ± SD.

* *P* < 0.05 compared to immediate postoperative.

^#^ *P* < 0.05 compared to Control group.

### 3.5 TPE improves the short-term prognosis of AMI patients after primary PCI

**Figure 2:**
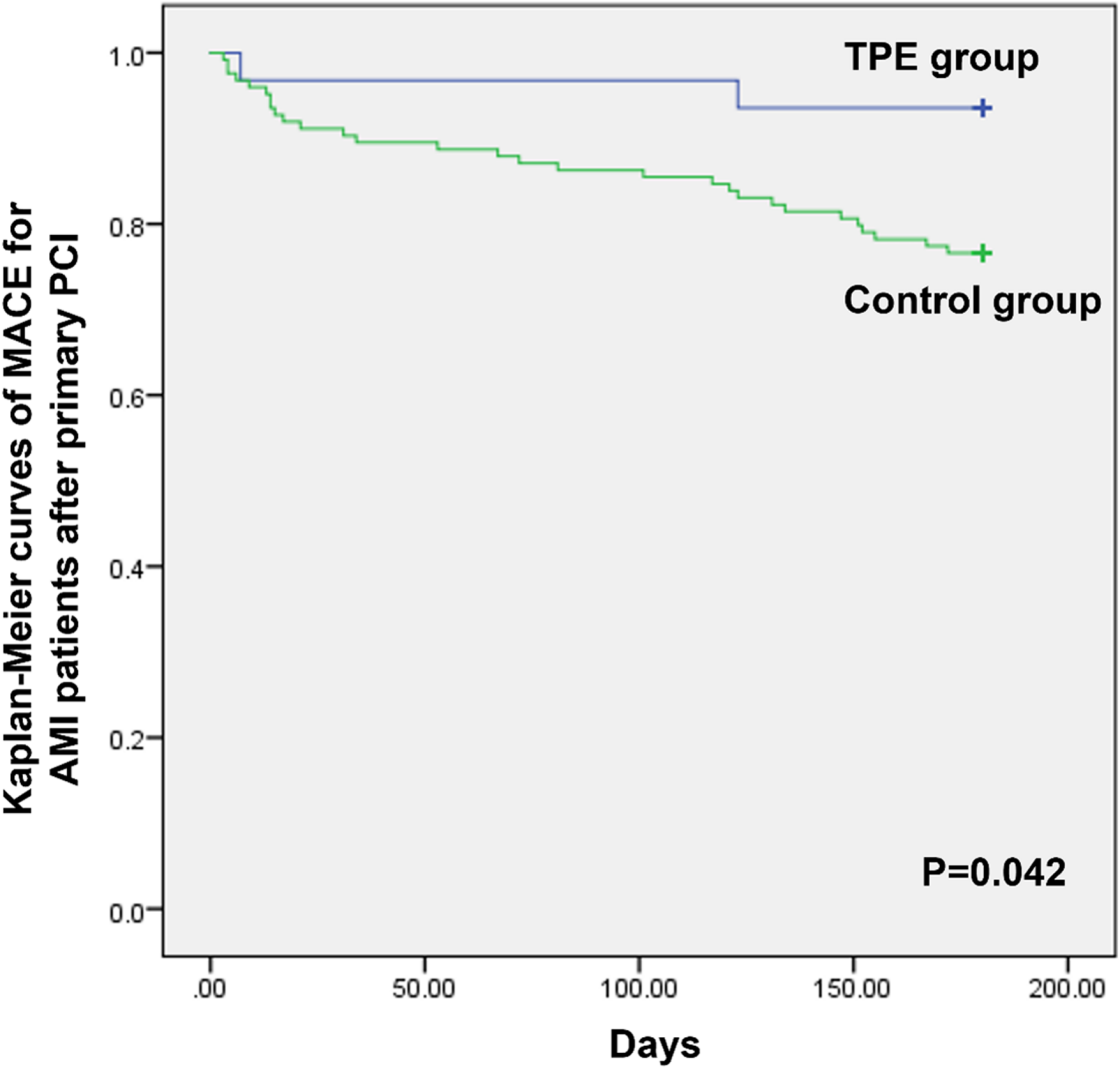
Kaplan-Meier curves of MACEs for patients with AMI after primary PCI between the TPE and control groups.

Finally, we compared the short-term clinical outcomes between the two groups at 6 months follow-up. There were no cardiac deaths in either group at 6 months. In the control group, 10, 15, and four cases of new heart failure, intractable angina, and recurrent MI, respectively, were reported, while one case of new heart failure and one case of intractable angina were reported in patients with AMI who received TPE. As shown in Figure 2, Kaplan-Meier curve analysis indicated that patients with AMI who received TPE had significantly lower rates of MACEs than the control group at 6 months follow-up (*P* = 0.042).

## 4 Discussion

Coronary microcirculation is defined as a complex network composed of pre-arterioles (≤500 μm in diameter), arterioles (≤200 μm in diameter), and capillaries and is regarded as the place of exchange for oxygen, nutrients, and metabolites in the heart (13, 14). Microcirculatory dysfunction results from multiple pathological changes, including smooth muscle cell dysfunction, microvascular remodeling, vascular rarefaction, microthrombosis, inflammation, and endothelial dysfunction (15). It has been reported that over 50% of patients without flow-limiting coronary lesions have functional abnormalities in coronary microcirculation, leading to an inadequate increase in blood flow to match myocardial oxygen needs and showing symptoms of typical angina (16). Microcirculatory dysfunction also occurs after reperfusion treatment among most patients with AMI and has been confirmed to be related to a higher risk of cardiovascular events in these patients, which is an intractable clinical problem (17). The existing coronary microcirculation treatment drugs are mainly statins, antiplatelet drugs, angiotensin converting enzyme inhibitor, β-blockers and nitrates. However, there is no consistent research result on the efficacy of these therapies (18). Treament of microcirculatory dysfunction still lacks pertinence and has insufficient evidence.

*Trichosanthes pericarpium*, first documented in a pharmaceutical monograph from the Northern and Southern Dynasties (420–589), is a widely used traditional Chinese medicine for the treatment of precordial pain. The extract of *Trichosanthes pericarpium* has now been widely utilized as an adjuvant therapy to improve blood circulation and resolve stasis in patients with coronary heart diseases in China. Previous phytochemical investigations of *Trichosanthes pericarpium* have reported the discovery of alkaloids, flavonoids,terpenoids and sterols. Its 70% ethanol extract have also been reported to contain at least 21 compounds (10). Recent basic scientific research suggests the following:

*Trichosanthes pericarpium*-derived alkaloids could inhibit platelet aggregation induced by adenosine diphosphate and arachidonic acid in rabbits while reducing the maximum platelet aggregation rate and inhibiting thrombosis formation (8). In vitro, Zhang et al. investigated the effects of TPE on hypoxia/reoxygenation (H/R)-induced injury in H9c2 cardiomyocytes and found that pretreatment with TPE significantly enhanced cell viability, increased the ratio of BCL-2/BAX, decreased apoptosis, and attenuated LDH release under H/R-induced injury conditions. One possible mechanism underlying the beneficial effects of TPE may involve the activation of the PI3K-AKT-NO signaling pathway in cardiomyocytes (19). Inspired by these findings, we investigated the influence of PCI on microcirculation and outcomes in patients with AMI.

The CTFC and IMR are two of the main invasive assessments to measure coronary microcirculatory dysfunction quantitatively (20–23). In the present study, we retrospectively enrolled 155 AMI patients with complete angiographic results of primary PCI and a 6-month follow-up including these two assessments and who were treated with TPE or not. The results of our study suggest that TPE could markedly reduce CTFC and IMR 6 months after primary PCI. The improving effects of TPE in early postoperative period on the microcirculatory inflammation and endothelial function may be important factors that lead to these phenomenoa.TPE was also observed to preserve cardiac function, attenuate the trend of cardiac hypertrophy, and improve short-term prognosis. Our study provides a new reference for applying traditional Chinese medicine in therapeutic strategies for microcirculatory dysfunction in patients with AMI.

This study has some limitations. First, this was a single-center retrospective study with small sample size. Limited by some study conditions, we adopted a retrospective case-control study to ensure that the study could include as many cases as possible. A large, prospective, randomized, controlled study using all-cause mortality as the endpoint is required to verify our conclusions. Second, the active constituent of *Trichosanthes pericarpium*, which has medicinal benefits in microcirculation, remains unclear. A recent study identified that *Trichosanthes pericarpium* contains 36 components, including amino acids, alkaloids, and nucleotides (24). Future studies should further screen and purify the key molecules in these components to achieve more precise treatment for patients with AMI.

In conclusion, in the context of standard treatment, TPE further improved coronary microcirculation, increased cardiac function, and reduced the rate of short-term MACEs. Our data suggest that TPE could be used in combination therapy for patients with AMI after primary PCI.

## Data Availability

The raw data supporting the conclusions of this article will be made available by the authors, without undue reservation.
